# Supplementary material for: Chromothripsis during telomere crisis is independent of NHEJ, and consistent with a replicative origin
Source: Genome Res. 2019 May;29(5):737–49. doi: 10.1101/gr.240705.118 (PMC6499312; doi:10.1101/gr.240705.118)
Supplement: Supplemental Material [file supp_gr.240705.118_Supplemental_file_1.zip › contigs/annotated_contigs/DB53/contig.14.DB53_length_1501_mean_cov_28.9207195203.docx]

**DB53_length_1501_mean_cov_28.9207195203**

```
CATTTGGTGATGAACATACAGTGATTCAAATATAACTTGGAATGTTTTAATATTTTTGGTTCTTTGGATTATTCATTTGAAAATACTCC
 >chr8:85466387-85466783 - E=6e-226 p=3e-02
TTACATAGACATGTGGCTCCTAGATTTGTAATATTTATATTTTCATATAACAGAAACACTAGACAACAATTAACACCATTATTTTGATA

TCTTTTCTCTTCAAAAGATGCACTCTCATGTTTAAGAGAACATGTTCTGGTTGGAAAACAAAACACAATGAACTACCGTAAATTGTTAC

ATCTCCAGTGTTTAAATGTGTGATAGTCTAATTAACTTAAAGTGAAAAATATGGAGATGACTTGAGCTAATGATAGAACATTGTTGCCT

ACACACGAGTAAATGTCTTTCAGCCATGATTACTTAATTT|G|TCCTGACTGAAATTCATTCTAGATCTGATGACAGTTAAGATATTCC
                                          >chr8:79142289-79142430 + E=1e-72 p=1e-02
TAGAGCTGATCGGCAGTGACTTTCTTCCACCGCTCTTAGTTAAATTAAAAAATAAGATGATATAAGTTAGTCATATTGTCCTTCGTGAA

CAGGAT|ATTTATGAATTTACACTCTT|TATGTTATTTCCTACAGATGTGTTCATGCAGAGAATAAGTTGATTTAATGCAATTATCTTA
                           >chr8:85358785-85358867 - E=3e-37
TTAAACAGATGATAAAATT|GA|ATTCTATACAGTTGTTGTTACATTACTCAAAACAGTGGATGAACAAACTGTCTAGAACTAGCAG|C
                    >chr8:78552423-78552489 - E=1e-27
CCATTTCTAACTTTAAGGAGTGCAATTTATGTGCTATGAAAGGACAA|CAGACTTACCCAAACCAGGGGATAATTAAATGGTTAGTAGC
 >chr8:79142239-79142286 + E=4e-16               >chr8:81118247-81118296 + E=2e-17
CTGTACTC|CT|GGGCCAAGCTGCTGCATCCTTATGTTCTCAGCCAGAGAAACAGCCTAGCAAACGCATCCAATGTGGCCTTTCCTCTG
           >chr17:68554213-68554309 - E=1e-45
AATTGGATACCCTGCC|ACA|AGTTTGCCACCAGATGTGCAGATAT|AAGAATACATTTGGGTGATTTGATGAGGACCAAGTAGTGAAT
                 >chr17:68553845-68553873 - E=1e-04 p=2e-02
ATGGGTGTT|CCTACTTTTAAATTTTTATTTAACAAATATTGGATATGGCACAGCCATAGTTGCTGAAGAATCCTGCTTACATTAAAAC
          >chr8:79152402-79152483 - E=1e-36
AT|GTCAGTGACACCAAGGTTCTAAAA|CT|GTACAGCACGTTACTGTACTGAATACTGTAGGCAATTGTAAAACAATGATAACTATTT
 >chr17:68800146-68800174 - E=1e-04  >chr8:78555650-78555727 - E=3e-34
GTGTATCTAAACATGTC|ATTCATTACG|AATGAATAATATATTCCTTAGATTAGCACTAATCTAGTT|CATTTGG|TTCCTACAAAAG
                            >chr8:81611264-81611303 + E=2e-11             >chr17:6855
CTACTCCATAAAACTG|TCCCTTTCAGTTTTA|TGAATTGGCAAATATTGACTCTAGCCATCCTAATTTGTTATCCTTTTAGGCTCCTA
3879-68553907 - E=1e-04         >chr8:81119158-81119403 + E=4e-132
TGGGTCCATTTTAGATATTTTTGGTTGTGTTGATTTTATTTCTTTTTGCTCCATTATATTGCTTCATTTCCCCTTTGTTTTTCCTTTAT

CCCTCTTTGATGACTCATCTTCATGCTATTCGGCCAGAATCTGGGTCTGGAGGAGTGTGAACCATGGAGGGTTTACATTATAAACAAGA

CATCGCCTCTC
```
